# A Green and Simple Protocol for Extraction and Application of a Peroxidase-Rich Enzymatic Extract

**DOI:** 10.3390/mps3020025

**Published:** 2020-03-26

**Authors:** Gonçalo P. Rosa, Maria do Carmo Barreto, Diana C. G. A. Pinto, Ana M. L. Seca

**Affiliations:** 1 cE3c—Centre for Ecology, Evolution and Environmental Changes/Azorean Biodiversity Group & Faculty of Sciences and Technology, University of Azores, Rua Mãe de Deus, 9500-321 Ponta Delgada, Portugal; goncalo.p.rosa@uac.pt (G.P.R.); maria.cr.barreto@uac.pt (M.d.C.B.); 2 LAQV-REQUIMTE, Department of Chemistry, University of Aveiro, 3810-193 Aveiro, Portugal; diana@ua.pt

**Keywords:** *Brassica rapa*, extraction, enzymatic extract, biocatalysis, peroxidase

## Abstract

Recently there is a great social expectation that scientists should produce more sustainable and environmentally friendly chemical processes. Within this necessity, biocatalysis presents many attractive features because reactions are often performed in water, under mild conditions, the catalyst is biodegradable and can be obtained from renewable raw materials. In this work, we propose a simple, rapid and low-cost method for the preparation and application of an enzymatic extract from turnip root. The protocol described includes (1) the preparation of the enzymatic extract, (2) the procedure for the assessment of the more favorable working parameters (temperature, pH) and (3) the methodology for the application of the extract as the catalyst for biotransformation reactions. We anticipate that the protocol in this research will provide a simple way for obtaining an enzymatic extract which can operate efficiently under mild conditions and can effectively catalyze the biotransformation of simple phenols.

## 1. Introduction

Recently, there is a higher societal awareness regarding the environmental questions of human activities, and science is no exception [1]. This increasing expectation that scientists should improve processes to become greener and more sustainable, led to the development of green chemistry. This term, postulated by Anastas and Warner [2], aims toward developing new products and processes that are less dangerous to human health and the environment, by eliminating or reducing the use and production of harmful substances as well as reducing harmful or toxic intermediates [3]. Basic principles of green chemistry cover a wide spectrum of organic synthesis methodology such as designing processes of organic synthesis to reduce byproduct/waste generation, reduce the use of hazardous chemicals/raw materials and enhance the use of safer or more environmentally-safe solvents and renewable raw materials. In addition, green chemistry promotes the best form of waste treatment, designing the process of chemical residues degradation, all following pollution prevention and sustainable development procedures, also focusing on energetic efficiency [4].

Within these principles comes catalysis, which has many attractive features in the context of green chemistry. Catalysts and their associated catalytic reactions could be homogeneous, heterogeneous or use enzymatic (bio-) catalysis. In homogeneous catalysis, the catalyst and the reactants are in the same phase [5]. Homogeneous catalysts apply to reactions in the gas phase or mostly in the liquid phase. Although these catalysts have some advantages such as excellent selectivity and single active sites, they suffer from some disadvantages, like poor thermal stability and difficult and high cost catalyst recovery [5]. Heterogeneous catalysts typically involve the use of solid catalysts placed in a liquid or gas reaction mixture [6]. Since they are in a different phase, heterogeneous catalysts can be easily separated from a reaction mixture, allowing the effective recovery and recycling of the catalyst, which is quite eco-friendly. Furthermore, this type of catalysts is very stable, maintaining the activity even under harsh reaction conditions [6]. Despite such advantages, heterogeneous catalysis has been thought to allow for less interaction and controllability than homogeneous catalysis due to a poor understanding of its reaction process [6]. With biocatalysts, the reactions are often performed in water, under mild conditions of temperature, pressure and pH [7,8]. Furthermore, the catalyst (an enzyme) is biodegradable and could be derived from renewable raw materials. The enzyme often affords high chemo-, regio- and stereoselectivities and avoids the need for protection and deprotection sequences which are required in classical synthesis methodology [1,9]. Some factors limit the industrial applications of enzymes, namely low long-term operational stability, in addition to the technically challenging process of recovery and reuse of the enzyme. In order to render enzyme utilization in biotechnological processes more favorable, different methods for cost reduction have been put into practice, one of which is immobilization [10]. The term “immobilized enzymes” refers to “enzymes physically confined or localized in a certain defined region of space with retention of their catalytic activities, and which can be used repeatedly and continuously” [11]. In addition to a more convenient handling of the enzyme, it also substantially simplifies the control of the reaction process [12] while enhancing the stability of the enzyme under both storage and operational conditions. Immobilization provides also an easy separation of the enzyme from the product, hence protein contamination of the product is minimized or even avoided [13]. Apart from simplifying the separation of the enzyme from the reaction mixture, enzyme immobilization in a matrix, like alginate, also remarkably reduces the cost of enzyme and consequently of the enzymatic products [10], since it usually allows using the enzyme for a longer period. However, enzymatic immobilization presents also some disadvantages like lower enzyme activity compared to native enzyme, additional costs with the matrix used for immobilization and lower reaction rates compared to native enzymes [14].

Biotransformation of chemical compounds usually resorts to using unpurified enzymatic extracts, since the associated cost is not too high, although these extracts only contain approximately 1%–30% of the pretended enzyme, besides other active and/or inactive enzymes and cell constituents. Additionally, enzymatic extracts are often more stable than purified enzymes due to the presence of a mixture of components that confer protection against undue oxidation and denaturation [7].

A group of enzymes often used in these procedures are peroxidases, which, due to their versatility and to their ubiquitous nature, have immense potential to be employed as industrial enzymes [15]. They catalyze the redox reaction for a wide range of substrates, which leads to the classification of peroxidases as an important group of enzymes for medicinal, biochemical, immunological, biotechnological and industrial applications. They have been successfully used for biopulping and biobleaching in the paper and textile industries [16,17]. Additionally, peroxidases extracted from different sources have been used for bioremediation and decolorization reactions [18,19,20,21]. They have been able to efficiently remove phenol derivatives from wastewater, like phenol, 2-chlorophenol, *m*-cresol, by oxidative polymerization, with 95% removal efficiency when associated with polyethylene glycol, and without needing further purification of the extract, which greatly reduces the associated costs [21]. Purified peroxidases have also been used in organic synthesis, bioremediation, as well as various analytical applications. For example, peroxidase-based biosensors find application in analytical systems for determination of hydrogen peroxide, glucose, alcohols, glutamate and choline [22]. Interesting works on the production of recombinant peroxidases in several hosts have been published so far, searching for a way to obtain a homogeneous and high yield source of these enzymes. Nevertheless, it has to be pointed out that the production of these recombinant enzymes still has not achieved the desired yields [23]. In *Escherichia coli*, the host where higher yields have been obtained, the enzymes are often found as protein aggregates which require laborious solubilization and refolding steps in order to obtain activity, with yields that make this production not competitive [24]. For most biotechnological and industrial purposes, therefore, the use of vegetable peroxidase sources is so far the more economic option.

In this regard, the present work aims to present a methodology for the simple, rapid and low-cost preparation of a peroxidase-rich enzymatic extract suitable not only for application in biotransformation reactions but also for application for educational purposes. In fact, the proposed protocol can be used in laboratory classes so that students acquire knowledge on how to obtain an enzyme extract and understand the concept of enzyme activity and some of the factors on which that activity depends.

## 2. Experimental Design

The protocol in this study is designed for the rapid preparation of a peroxidase-rich enzymatic extract from turnip roots (*Brassica rapa* var. *rapa* Barkant), obtained from a local market, which will be ready to be applied as a catalyst in biotransformation reactions. The turnip root was chosen due to availability, but the protocol described can be used for other peroxidase sources [25,26,27,28,29].

In a first stage, the steps for obtaining the extract are presented, then the method for determining the extract’s enzymatic activity and optimizations are discussed, and finally, the procedure for the applying the extract as a catalyst in a biotransformation of guaiacol is explained.

### 2.1. Materials

#### 2.1.1. Preparation of Enzymatic Extract

- Fresh turnip (*Brassica rapa* var. *rapa* Barkant) roots.
- Phosphate buffer 100 mM pH 6.5.
- Cotton wool.

#### 2.1.2. Protein Quantification

- Bradford Reagent
- Bovine Serum Albumin (BSA) (Sigma, Darmstadt, Germany)

#### 2.1.3. Biotransformation Reaction

- 2,2′-Azino-bis(3-ethylbenzothiazoline-6-sulfonic acid) diammonium salt (ABTS) (Sigma, Darmstadt, Germany).
- Phosphate buffer 300 mM pH 5.5, 6.5 and 7.5.
- Acetate buffer 300 mM pH 3.5 and 4.5.
- Hydrogen peroxide (H_2_O_2_) (Fluka, Charlotte, NC, USA).
- Dimethyl sulfoxide (DMSO) (Sigma, Darmstadt, Germany).
- Acetone (Sigma, Darmstadt, Germany).
- Guaiacol (2-methoxyphenol) (Sigma, Darmstadt, Germany).

### 2.2. Equipment

- Eppendorf 5810R centrifuge (Eppendorf—Hamburg, Germany).
- EVOLUTION 160 UV–Vis Thermo Scientific thermostated spectrophotometer (Thermo Fisher Scientific, Waltham, MA, USA).

## 3. Procedure

### 3.1. Preparation of Enzymatic Extract. Time for Completion: 00:15 h

1. Wash and peel the turnip roots.
2. Dice 20 g of the peeled turnip roots.
3. Add 100 mL of cold phosphate buffer 100 mM, pH 6.5 to the diced roots.
4. Homogenize the mixture, immersed in ice using a commercial blender, until no lumps are observed.
5. Filter the homogenized mixture with cotton wool to remove suspended fibrous solid particles.
6. Centrifuge the filtrate at 18,514× *g* for 5 min at 4 °C.
7. Collect the supernatant and store it in ice.
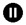
**PAUSE STEP** If the extract prepared is not going to be readily used, it can be stored at −80 °C without loss of activity.

### 3.2. Protein Quantification—Bradford Assay. Time for Completion: 00:30 h

This assay was performed based on Bradford test [30].

1. Prepare BSA dilutions for standards (from 100–1000 µg/mL).
2. Use phosphate buffer 100 mM, pH 6.5 as a blank (0 µg/mL).
3. Mix 2500 µL of Bradford protein-dye solution with standards or enzymatic extract samples.
4. Incubate for 10 min.
5. Measure the absorbance at 595 nm.
6. Plot a concentration/absorbance curve with the values obtained for standards.
7. Substitute the absorbance values of the extract samples on the equation of the curve to obtain the total protein concentration.

### 3.3. Biotransformation Reaction

#### 3.3.1. Assessment of Extract Activity. Time for Completion: 00:05 h

1. On two 3 mL cuvettes, add 2150 μL of acetate buffer 300 mM (pH 4.5), each.
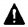
**CRITICAL STEP** All reagents and samples need to be brought to 35 °C in advance, to prevent temperature fluctuations during the test.
2. Proceed to add 100 μL ABTS (0.7 mM in acetate buffer 300 mM pH 4.5) to both cuvettes.
  **OPTIONAL STEP** Other colored substrates can be used, as long as they react with the enzymatic extract, resulting in a change in absorbance.
3. Add 50 μL of H_2_O_2_ 0.3% (v/v) in acetate buffer 300 mM (pH 4.5) to both cuvettes and mix them thoroughly.
4. Put the cuvette in the thermostated spectrophotometer, with the temperature set to 35 °C, and let it remain there for 2–3 min, to reach temperature equilibrium.
5. To the first cuvette, add 50 μL of acetate buffer 300 mM (pH 4.5), and set the blank in the spectrophotometer, at 414 nm, with the temperature set to 35 °C.
6. To the remaining cuvette, add 50 μL of the enzymatic extract and shake the mixture.
7. Immediately after the addition of the extract, start registering the absorbance in the thermostated spectrophotometer, always with the temperature set to 35 °C.
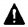
**CRITICAL STEP** To obtain a more correct assessment of the enzymatic activity, the absorbance has to be registered from the moment the reaction starts, so step 6 must be done in the fastest way possible.
8. Follow the absorbance of the sample at 414 nm, for 90 s.
9. With the data obtained, plot a curve which will allows calculating the enzymatic activity of the extract.
  **OPTIONAL STEP** All the reaction parameters (temperature, pH, etc.) described are optimal for this enzymatic extract activity to the substrate ABTS. However, if there is the need to test the effect of changing these parameters in the activity of the enzymatic extract or, if a different substrate is used, the working parameters could be reassessed using the same methodology used with ABTS.

#### 3.3.2. Biotransformation. Time for Completion: 48:00 h

1. Set a water bath to 35 °C.
2. Prepare 20 mL of substrate at a concentration of 7.25 mM in acetate buffer 300 mM (pH 4.5). Other volumes can be used, if needed.
  **OPTIONAL STEP** If the substrate is not soluble under these conditions, prepare the substrate with an appropriate solvent and add the buffer to obtain a mixture where the enzymatic extract retains if not all, at least a % of its activity. This proportion can be assessed by the procedure described in Section 3.3.1.
3. Gradually add 0.4 mL of H_2_O_2_ (9.43 mM) to the reaction mixture every 8 h, to a final volume of 2.4 mL.
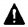
**CRITICAL STEP** The concentration of H_2_O_2_ is higher than substrate concentration to prevent H_2_O_2_ from becoming a limiting step. The addition of this reactive should be performed in small portions during the reaction, rather than adding all in the beginning. This is due to the fact that adding a large amount at once may cause the decomposition of the prosthetic heme and also modification of the higher structure of peroxidase, both essential for enzyme activity [31].
  **OPTION** Other time intervals for adding H_2_O_2_ can be chosen, depending on the laboratory equipment to ensure the gradual and fractionated addition of the reactive, like peristaltic pumps.
4. Start the reaction by adding 750 μL of turnip enzymatic extract and repeat every 12 h, to ensure that there are always active molecules of the enzyme in the reaction medium.
  **OPTION** The addition of enzymatic extract and H_2_O_2_ can be done simultaneously, but it also depends on laboratory equipment. In fact, in this work the conditions chosen were limited by only one peristaltic pump with a timer, used to the hydrogen peroxide addition, while enzyme addition was performed manually. Considering that in the blank reaction, performed with peroxide but not enzymatic extract, there were no changes in the reactional medium, the conditions chosen did not affect the development of the catalytic reaction.
5. Stop the reaction after 48 h.

## 4. Expected Results

### 4.1. Properties of Enzymatic Extract

The described extraction process is a very simple procedure to obtain a ready-to-use turnip enzymatic extract. However, it is important to assess some properties of this extract. Thus, it is necessary to verify if it is, in fact, an extract that exhibits enzymatic activity, to determine what is the protein content present and the variability of these parameters between extractions.

The results show that, besides being a quite fast procedure, it also yielded an extract with good amounts of protein per mL, and good total peroxidase activity (Table 1). In fact, when compared with similar works from the literature, the presented method has the advantage of being the one where the extract is obtained in fewest time, with more activity and with less intermediate steps [32,33].

The total protein values determined were virtually unchanged, in various extractions performed with roots obtained in autumn. For turnip roots obtained in the winter, the total amount of protein present in the extract was much lower (Table 1).

These variations in total protein concentration were reflected in the total activity (U) of the extract, with the extracts prepared with roots collected in winter, being less active than the ones with the roots collected in autumn (Table 1). This variability has been described for turnip peroxidase, where it was found that the concentration and activity of this enzyme varies seasonally, depending on the development stage [34]. In this regard, it is recommended that large amounts of extract should be prepared in seasons where it is more active and, stored at −80 °C to further usage.

### 4.2. Assessment of Extract Activity in Different Conditions

The assessment of extract activity in different conditions is important for the determination of which conditions are more suitable for the biotransformation reactions, and also, in case that some problems appear during the application on the biotransformation reaction, to verify which adjustments can be made, without deeply affecting the activity of the enzymatic extract.

In this stage, it is possible to test the optimal temperature, pH, the stability of the enzymatic extract when exposed to certain temperatures for long periods or the effect of solvents in its activity. Only with this information it is possible to choose the most suitable parameters for the biotransformation reaction.

**Figure 1:**
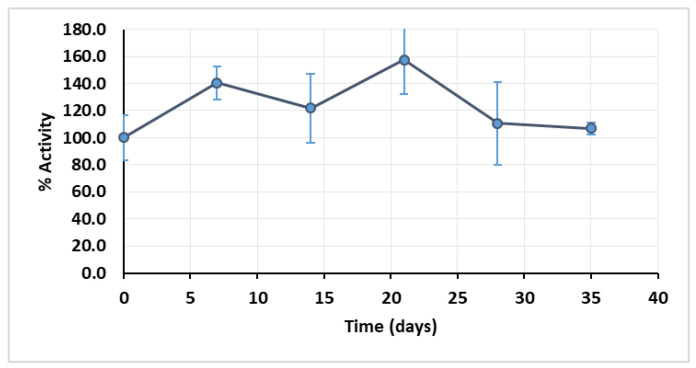
Curve of enzymatic extract stability when stored at −80 °C; pH 4.5. Error bars represent standard deviation.

The first parameter to be tested was the storage stability of the extract, because of seasonal loss of protein content and activity reported above. The extract was stored at −80 °C and its activity assessed every 7 days until the 35th day. The extract retained full activity after 35 days, and it was observed that during that period of storage, namely between the 7th and the 21th day, the extract activity was higher than the registered in day 0 (Figure 1).

This is most likely because the freezing process destroys some cellular structures that were preserved during the extraction process, which could be encasing part of the peroxidase, therefore contributing to a higher availability of the free enzyme and consequently to an increased reaction rate.

**Figure 2:**
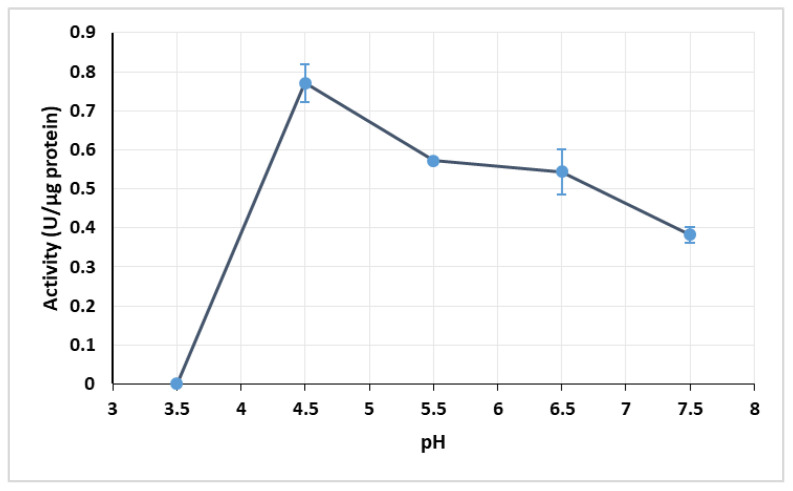
Effect of pH on enzymatic extract activity at 30 °C. Error bars represent standard deviation.

The optimal operating pH was found to be pH 4.5, with an activity of 0.772 U/µg protein. The other pH values tested yielded significantly lower activities, with the lowest activity being obtained at pH 3.5. The low activity obtained at pH 3.5 might be related with enzyme inactivation caused by the acidic conditions, since low pH usually causes the denaturation of proteins, leading to their loss of function. There was no significant difference between the enzyme activity at pH 5.5 and 6.5 (Figure 2).

**Figure 3:**
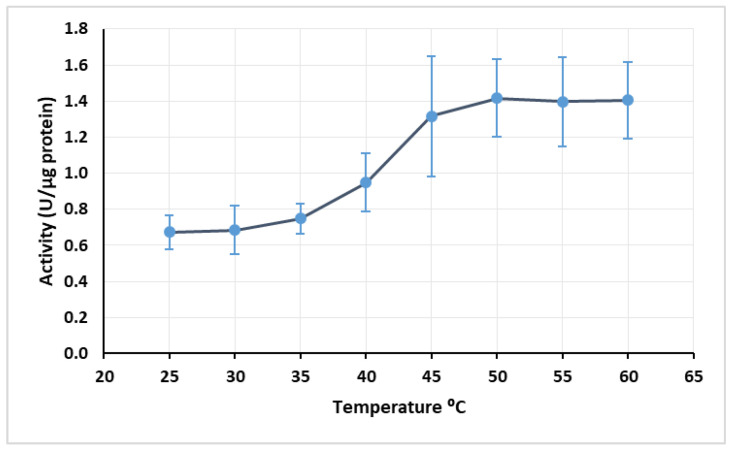
Effect of temperature on enzymatic extract activity at pH 4.5. Error bars represent standard deviation.

Regarding temperature, the maximum activity was achieved at 50 °C with 1.416 U/µg protein, although the difference of activity from 45 to 60 °C was not too pronounced (Figure 3).

The optimal temperature for the extract activity was 10 °C higher than the one obtained by Motamed et al. [32]. This may be due to the fact that in the cited work the enzyme was semi-pure, and in this work, the enzyme was in an extract together with other constituents. The presence of other constituents protects the enzyme against denaturation, which may explain why the enzymatic extract resisted higher temperatures than the semi-pure enzyme studied by Motamed et al. [32]. 

**Figure 4:**
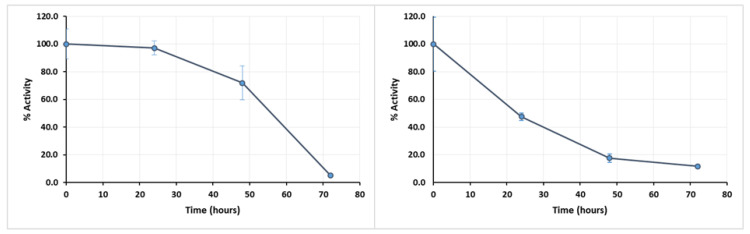
Curve of long-term enzymatic extract thermostability for 72 h, at pH: 4.5 and 30 °C (left panel) or 40 °C (right panel). Error bars represent standard deviation.

To assess the stability of the extract over long-term exposure to temperature, the enzymatic extract was incubated during 72 h at different temperatures, with the activity being assessed every 24 h. After 24 h, the activity of the extract was almost null (data are not shown) at the higher temperatures. Even when incubated at 40 °C, a severe activity loss was seen after 24 h, while at 30 °C the tendency for the activity to decrease only became most significant after 48 h (Figure 4).

With these results, it became evident that the enzymatic extract would not remain active in longer-term reactions at 40 °C. However, the enzymatic extract activity is lower at 30 °C, as observable in Figure 3. In this regard, the temperature of 35 °C was elected the most suitable for long-time reactions, in an attempt to maintain a compromise between extract activity and its stability.

**Figure 5:**
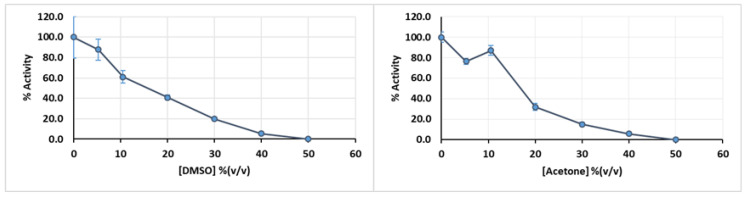
Effect of DMSO (left panel) or acetone (right panel) in enzymatic extract activity at pH 4.5. Error bars represent standard deviation.

Sometimes, the intended substrate is not soluble in the buffer used as a reaction medium for the enzyme. In this case, it might be necessary to prepare the substrate with an appropriate solvent and add the buffer to obtain a mixture where the enzymatic extract retains if not all, at least a significant fraction of its total activity. In the specific case of the turnip enzymatic extract, it retains about 40% of its activity when exposed to a concentration of 20% (v/v) DMSO (Figure 5, left panel) and 35% when exposed to same concentration of acetone (Figure 5, right panel), so either one of these solvents could be selected depending on substrate solubility, without complete loss of activity.

### 4.3. Biotransformation Reaction

To demonstrate the expected results when the enzymatic extract is applied on biotransformation reactions performed as described in point Section 3.3.2, it was selected the phenolic compound guaiacol, a substrate that had been previously biotransformed with a peroxidase enzyme [35], which would allow a better assessment of the efficacy of the enzymatic extract obtained by the proposed protocol.

**Figure 6:**
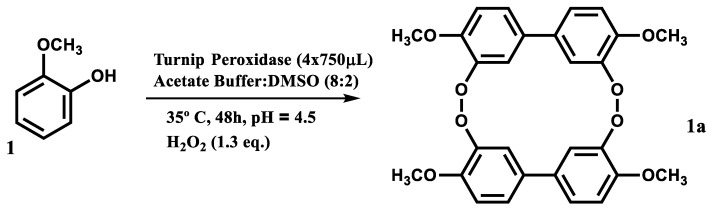
Peroxidase-catalyzed transformation of guaiacol (**1**) to tetraguaiacol (**1a**).

According to the literature, the reaction of guaiacol with hydrogen peroxide catalyzed by a peroxidase should lead to the formation of tetraguaiacol (Figure 6(1a)), a tetramer of guaiacol.

**Figure 7:**
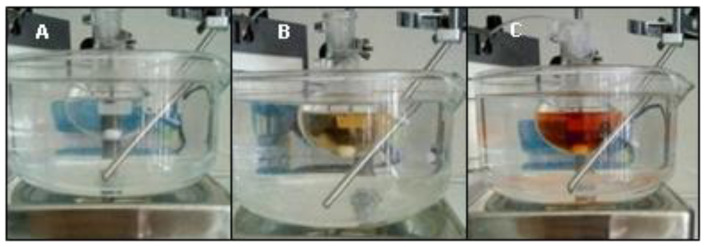
Biotransformation reaction: Development of the reaction using guaiacol catalyzed by the extract. (**A**) At 0 min; (**B**) at 5 min; (**C**) 10 min.

The biotransformation of guaiacol (Figure 6(1)) catalyzed by the enzymatic extract in the chosen reactional conditions (Figure 6) occurred very quickly and is easily observable even without spectrophotometric methods, as seen by the shift of color from transparent to amber (Figure 7).

A blank reaction was also performed, in the same conditions, but with the addition of phosphate buffer instead of the enzymatic extract. In this reaction no transformation was observed, which shows that the enzymatic extract was definitively responsible for the observed transformation and not a result of the direct reaction between the guaiacol and the H_2_O_2_.

Since changes in the chemical structure of phenolic compounds are often reflected in the UV/Vis absorption spectra, the reaction progression was followed by recording the absorption spectrum of reactional mixture over reaction time. The reaction progression can be also followed by other methods like zymography, high performance liquid chromatography (HPLC) or near-infrared spectroscopy [29,35,36], however, since the main goal of this protocol is to describe a simple and low-cost method, the authors opted for UV/Vis spectrophotometry.

As described in the literature [36], the tetraguaiacol (Figure 6(1a)) absorbs radiation between 400 and 500 nm and has its maximum absorbance at 470 nm. Thus, the absorbance level at this wavelength over the reaction time is used as a marker of guaiacol peroxidation.

**Figure 8:**
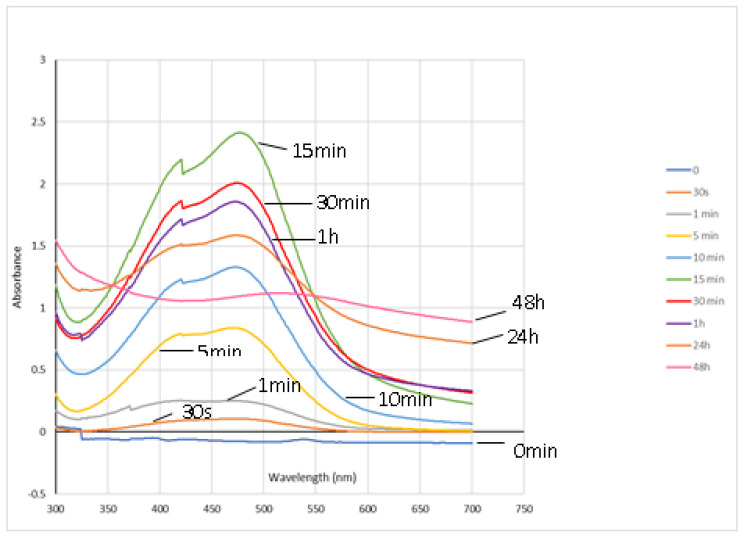
Visible spectrophotometry of guaiacol oxidation products during reaction.

In Figure 8 it is observable that only 1 min after starting the reaction, there is an increase of the absorbance around the 470 nm, meaning that some transformations have occurred. From 1 min, the absorbance at 470 nm increases significantly and reaches its maximum value at 15 min which means that tetraguaiacol (Figure 6(1a)) concentration achieves its maximum.

Between 30 min and 48 h, the absorbance decreases, which could be explained either by the low stability of this product [37], or because, in the opinion of the authors, it could itself be a substrate to the enzyme. Despite that, this biotransformation reaction gave some important information: it proved that the reaction conditions chosen allow the enzymatic extract to work and to catalyze biotransformations, being effective in the transformation of simple phenols such as guaiacol.

## 5. Conclusions

In conclusion, the method described is a rapid procedure for the preparation of a ready-to-use peroxidase-rich enzymatic extract from turnip roots. It has the advantage of being versatile, since this same procedure can be employed to obtain peroxidase from sources other than turnip, In addition, there is no need for further purifications, which turns the process much cheaper. The extract obtained was applied in biocatalysis and the results obtained indicate that turnip peroxidase extract is effective in catalyzing the biotransformation of simple phenols, like guaiacol. The ability of the extract to polymerize simple phenols hints for a possible application in bioremediation, namely on the removal of phenol derivatives from wastewater. On the other hand, the simplicity of this protocol makes it useful for didactic purposes in laboratory classes, allowing to demonstrate how to obtain an enzymatic extract and the various factors on which the activity of an enzyme depends, as well as to verify the action of the enzyme in reactions with visual effect.

## Figures and Tables

**Table 1:** Total amount of protein present on the enzymatic extract obtained from turnip roots in different seasons.

Extraction	Season	Total Protein (μg/mL Extract)	Total Activity (U/μg Protein)
1	Autumn	349 ± 11	0.77 ± 0.05
2	Autumn	358 ± 15	0.81 ± 0.02
3	Autumn	353 ± 26	0.79 ± 0.06
4	Autumn	351 ± 18	0.80 ± 0.03
5	Winter	201 ± 6	0.54 ± 0.01
6	Winter	205 ± 6	0.57 ± 0.05
7	Winter	211 ± 13	0.60 ± 0.02
8	Winter	210 ± 4	0.59 ± 0.01
